# SGLT-2 inhibitors in peritoneal dialysis: A new dawn for cardio-renal protection

**DOI:** 10.1016/j.bbrep.2026.102878

**Published:** 2026-11-07

**Authors:** Yanling Song, Xiaofei Huang, Weijie Yuan, Nan Zhu

**Affiliations:** a Department of Nephrology, Shanghai Jiao Tong University School of Medicine Affiliated First People's Hospital, Shanghai, 200080, China; b Department of Nephrology, Jintan Affiliated Hospital of Jiangsu University, Changzhou, 213200, China

**Keywords:** Peritoneal dialysis, SGLT-2 inhibitors, Cardiovascular, Metabolism, Residual kidney function

## Abstract

The rising global incidence of chronic kidney disease has positioned peritoneal dialysis (PD) as a critical renal replacement therapy. However, its long-term efficacy is often compromised by peritoneal fibrosis and encapsulating peritoneal sclerosis, leading to treatment failure in kidney failure patients. Furthermore, this population faces compounded health challenges, including heightened cardiovascular risk and metabolic syndrome. Sodium-glucose co-transporter 2 (SGLT-2) inhibitors, initially developed as anti-diabetic agents, have garnered significant attention for their demonstrated clinical benefits in renal and cardiovascular protection, as well as metabolic improvement. This review aims to comprehensively analyze the mechanisms of action and clinical application prospects of SGLT-2 inhibitors in PD patients, synthesizing evidence from preclinical studies, observational data, and extrapolated findings from trials in chronic kidney disease (CKD) populations. By synthesizing existing literature, we seek to provide insights for clinical practice and illuminate future research directions.

## Highlights

• SGLT-2 inhibitors preserve residual kidney function in PD patients.
• They reduce peritoneal fibrosis and oxidative stress.
• Cardiovascular benefits support their use in high-risk PD patients.
• Clinical evidence indicates safety, but complication monitoring is warranted.
• Dedicated RCTs are needed to confirm long-term outcomes.

## 1 Introduction

Peritoneal dialysis (PD) is an important treatment for patients with kidney failure. Despite its widespread use, PD is associated with several challenges, the underlying mechanisms of which are not yet fully understood [1]. Sodium-glucose co-transporter-2 (SGLT-2) inhibitors are a relatively new class of anti-diabetic drugs that primarily work by reducing the reabsorption of glucose and sodium in the S1 segment of the proximal renal tubule [2], and have become a popular research focus due to their demonstrated cardiovascular and renal protective effects across a broad spectrum of patients, including those with and without diabetes.

A major concern in PD patients is the progressive decline in residual kidney function (RKF) [3]. SGLT-2 inhibitors are hypothesized to preserve RKF through various pathways [4]. Experimental studies have shown that SGLT-2 inhibitors can reduce the reabsorption of glucose, alleviate the high-glucose environment of the peritoneum, thereby improving the ultrafiltration effect of dialysate [5]. In addition, SGLT-2 inhibitors may also play a protective role by reducing peritoneal fibrosis and improving peritoneal function [6], potentially improving fluid management and reducing the risk of technique failure. These findings provide a theoretical basis for the application of SGLT-2 inhibitors in the PD population.

The well-documented cardioprotective properties of SGLT-2 inhibitors further strengthen their therapeutic potential in PD patients [7], a group inherently at an elevated risk for cardiovascular disease. Numerous studies have confirmed their role in reducing the incidence of major cardiovascular events [8], [9]. Large-scale clinical trials and meta-analyses have established SGLT-2 inhibitors as a cornerstone of cardio-renal protective therapy in CKD and type 2 diabetes mellitus (T2DM). Consequently, incorporating SGLT-2 inhibitors into the treatment regimen for PD patients could offer a dual benefit: preserving renal function while simultaneously lowering cardiovascular risk, ultimately contributing to improved survival rates [10].

In summary, SGLT-2 inhibitors hold promise for improving outcomes in peritoneal dialysis patients. This review synthesizes current evidence from preclinical studies and clinical data—including observational studies and extrapolated findings from CKD trials—to explore their potential mechanisms of action, clinical benefits, and safety considerations in the PD population.

## 2 Characteristics of peritoneal dialysis patients

Peritoneal dialysis is an effective treatment for end-stage kidney disease, especially for patients who are unable to receive kidney transplantation or hemodialysis [11]. PD patients exhibit distinct clinical characteristics that are intrinsically linked to the procedure's pathophysiology, its associated complications, and underlying cardio-renal risk factors.

### 2.1 Pathophysiology of peritoneal dialysis-related metabolic complications

As a renal replacement therapy for kidney failure [12], PD leverages the peritoneum as a semi-permeable membrane. It facilitates the removal of water and toxins by establishing an osmotic gradient between the blood and the dialysate, driving solute and fluid transfer from the vascular system into the peritoneal cavity [13], [14], Glucose from the dialysate diffuses into peritoneal capillaries, gradually dissipating this osmotic gradient and consequently reducing the ultrafiltration rate [14].

Human peritoneal mesothelial cells (HPMC) in the peritoneum are essential for maintaining peritoneal integrity and fluid balance and play a critical role in solute and water transport, immune regulation and inflammatory responses, and tissue repair [15], [16]. Chronic exposure to the high-glucose dialysate can inflict damage and lead to a loss of HPMCs [17]. Locally, this can disrupt peritoneal homeostasis, promote peritoneal fibrosis, impair the peritoneum's transport function for small molecules, and even lead to ultrafiltration failure [13], [18]. Systemically, absorption of glucose from the dialysate contributes to metabolic dysregulation, including hyperglycemia, dyslipidemia, insulin resistance, and weight gain, compounding the metabolic burden in PD patients. Interestingly, research has identified the presence of SGLT-2 in both the human peritoneum and HPMCs. Its expression appears to increase with the duration of PD and is markedly elevated in patients with encapsulating peritoneal sclerosis (EPS) [19].

In essence, sustained exposure to a high-glucose environment exerts both local effects—damaging peritoneal mesothelial cells and promoting fibrosis—and systemic effects—contributing to metabolic dysregulation. Both local and systemic consequences contribute to an increased risk of ultrafiltration failure and cardiovascular complications.

### 2.2 Challenges faced in peritoneal dialysis

Patients undergoing PD encounter a myriad of challenges. Ultrafiltration failure (UF) is defined as the failure to achieve a net ultrafiltration of at least 400 ml during a 4-h dwell with 4.25% glucose [20]. Long-term PD leads to changes in the peritoneum, the most important of which are complications such as peritoneal fibrosis and encapsulating peritoneal sclerosis [19]. According to research, encapsulating peritoneal sclerosis is a serious but rare complication of long-term peritoneal dialysis that almost always occurs in patients treated for more than 5 years [21].

Furthermore, the high concentration of glucose in peritoneal dialysate as an osmotic agent can lead to metabolic problems such as hyperglycemia and diabetes in patients [5]. Nutritional issues are also prevalent, with hypoalbuminemia and malnutrition frequently observed, further complicating metabolic management [22], [23]. Studies have shown that glucose absorption from dialysate not only affects blood glucose levels in patients but may also worsen dyslipidemia, insulin resistance, metabolic syndrome, weight gain, and peritoneal function, thereby affecting the effectiveness of dialysis [24], [25], [26], [27].

### 2.3 Cardio-renal risk factors

Mortality rates remain high among PD patients, with recent data indicating a 5-year survival rate of less than 50% after dialysis initiation [28]. Cardiovascular complications are the main cause, with 40% of deaths attributed to arrhythmias or cardiac arrest [28]. The high burden imposed by kidney function loss can explain the higher incidence and mortality of cardiovascular disease (CVD) in dialysis patients. As renal function declines, uremic toxins accumulate in the blood, disrupting cell function and causing cardiovascular damage [29]. Loss of residual kidney function (RKF) is one of the major risk factors for CVD in PD patients and increases the risk of all-cause mortality and CVD mortality [30]. Dialysis patients are at the highest risk of CVD and stand to benefit the most from effective cardioprotective therapy [31]. Numerous studies and meta-analyses have proven the cardio-renal protective effects of SGLT-2 inhibitors across diverse patient populations, and ongoing research is exploring their applicability in the PD setting.

## 3 Mechanism of action of SGLT-2 inhibitors

While the precise mechanisms by which SGLT-2 inhibitors confer benefits in PD patients are still under investigation and largely derived from preclinical models and studies in non-dialysis CKD populations, recent evidence suggests they act through multiple pathways. These include inhibiting epithelial-mesenchymal transition (EMT), enhancing mitochondrial function and energy metabolism, blocking pro-inflammatory signaling, mitigating glucotoxicity, and protecting the vascular endothelium. Beyond glycemic control, these pleiotropic effects underpin their significant potential for cardiovascular and renal protection in the PD setting (Fig. 1).

**Fig. 1:**
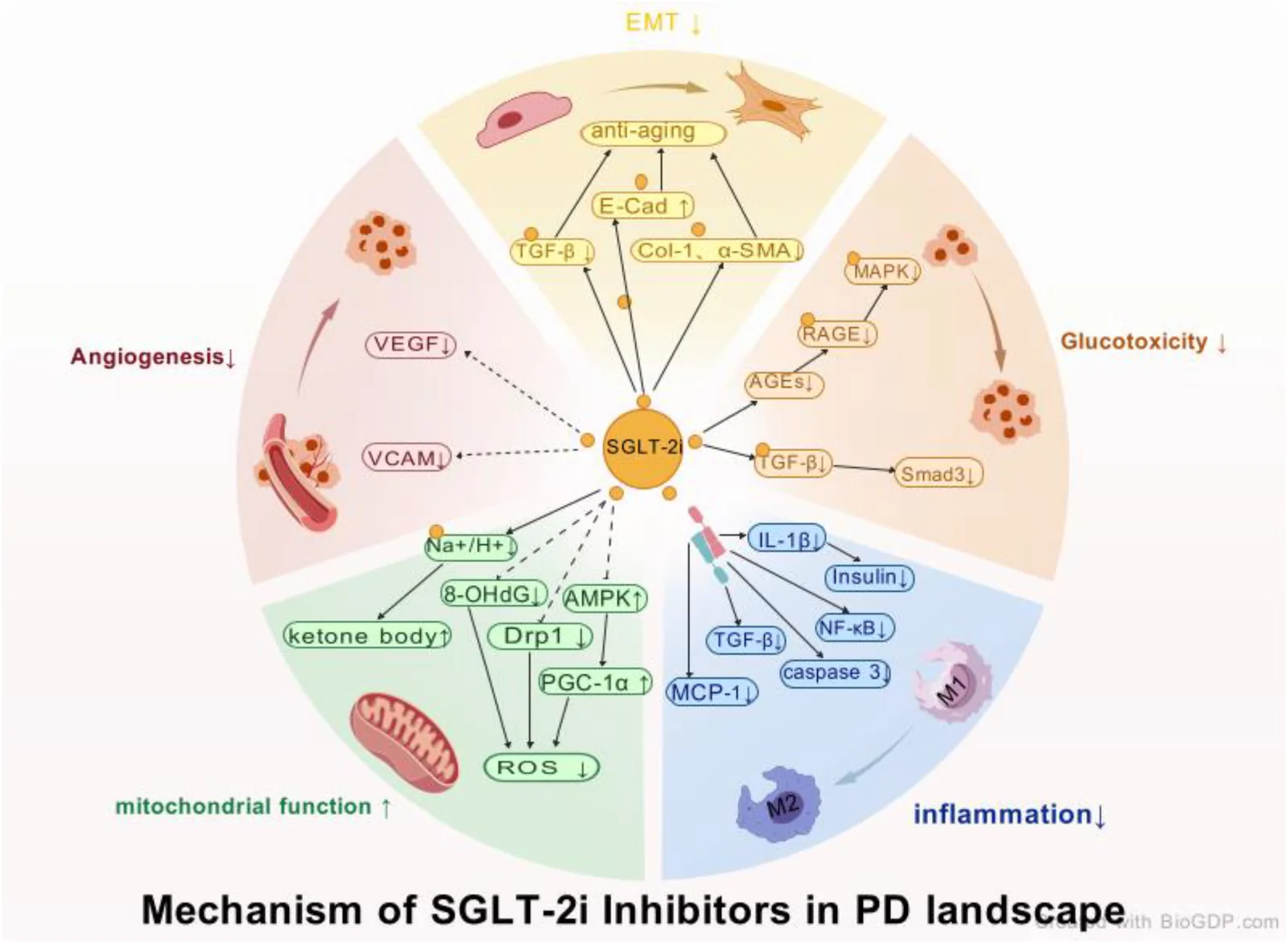
(Created with BioGDP.com): Mechanism of SGLT-2i Inhibitors in PD landscape. SGLT-2i,Sodium-glucose co-transporter 2 inhibitors; EMT, Epithelial-Mesenchymal Transition; E-cadherin, E-cad; TGF-β,transforming growth factor β; COL-1, collagen I; α-SMA, α-smooth muscle actin; ROS,reactive oxygen species; NF-κB, nuclear factor kB; AMPK, AMP-activated protein kinase; PGC-1α,Peroxisome proliferator-activated receptor-gamma coactivator-1α; Drp1,dynamin-1-like protein; 8-OHdG, tubular 8-hydroxy-2′-deoxyguanosine; IL-1β, interleukin 1β; MCP-1, monocyte chemoattractant protein1; M1, inflammatory macrophages; M2, anti-inflammatory macrophages; VEGF, Vascular Endothelial Growth Factor; VCAM, vascular adhesion molecules; AGEs, Advanced glycation end products; RAGE, receptor for AGEs; MAPK, Mitogen-Activated Protein Kinase; ↑,increased; ↓,suppressed.

### 3.1 Inhibition of epithelial-mesenchymal transition (EMT)

In the peritoneal microenvironment, the transdifferentiation of epithelial cells into motile mesenchymal cells is referred to as epithelial-mesenchymal transition [32]. Experimental evidence shows that a high-glucose environment and exposure to peritoneal dialysis solution (PDS) impair rat peritoneal mesothelial cells (RPMCs) and subcutaneous tissue, inducing EMT, which leads to reduced fluid exchange—a major complication of long-term peritoneal dialysis [33]. This local peritoneal process plays a primary role in fibrosis induction and subsequent deterioration of peritoneal function [34].

EMT is closely linked to cellular senescence, and studies suggest that anti-senescence strategies can suppress it [35]. Recent findings indicate that SGLT2 inhibitors may attenuate EMT in the context of PD by exerting anti-senescent effects [35], [36]. For instance, they have been shown to reduce markers of senescence and EMT, decrease reactive oxygen species (ROS) production, and alleviate DNA damage in glucose-treated mouse models [35]. Furthermore, experiments have demonstrated that empagliflozin suppresses the expression of Col-I and α-SMA in peritoneal fibrosis (PF) mice, increases E-cadherin levels, and reduces TGF-β1-induced EMT in human peritoneal mesothelial cells (HPMCs), ultimately delaying the progression of PF [37].

The transforming growth factor-beta (TGF-β) signaling pathway is a well-known inducer of EMT [32], [38], [39]. SGLT2 inhibitors have been proven to inhibit TGF-β, thereby suppressing EMT [39]. Therefore, SGLT2 inhibitors can reduce the aging of peritoneal mesothelial cells and inhibit EMT and peritoneal fibrosis in PD patients through mechanisms such as TGF-β suppression, thereby prolonging the duration of peritoneal dialysis. Studies have confirmed that peritoneal dysfunction accounts for approximately 30% of technical failures, and peritoneal fibrosis is one of the primary causes of peritoneal dysfunction in long-term PD [40]. This is supported by animal models where SGLT-2 inhibitors reduced dialysate-induced peritoneal thickening and collagen deposition.

### 3.2 Alleviating glucotoxicity injury

Within the peritoneal membrane, a functional peritoneum is essential for adequate ultrafiltration and fluid balance [41]. The glucotoxic environment itself can trigger harmful changes in HPMC, such as EMT, as well as pro-inflammatory and pro-fibrotic effects [42]. Glucotoxicity arises secondary to glucose degradation; this degradation alters the intracellular NADH/NAD + ratio, leading to pseudohypoxia, which triggers harmful changes in HPMC by stimulating the hypoxia-inducible factor-1 (HIF-1) gene [43].

SGLT-2 inhibitors intervene in these pathways. Empagliflozin significantly reduces the expression of the RAGE receptor in a high-glucose environment [44], inhibiting the AGEs-induced phosphorylation of ERK1/2 and p38 MAPK [45], Furthermore, SGLT-2 inhibitors can inhibit peritoneal fibrosis by blocking the AGEs-RAGE-MAPK axis [46], [47].

High glucose levels in dialysate also stimulate excessive TGF-β1 secretion, a key driver of peritoneal damage [40], partly through its interaction with macrophages [48]. SGLT2 inhibitors have been proven to inhibit TGF-β/Smad signaling, thereby suppressing fibrosis [37].

### 3.3 Protecting vascular endothelium

Within the peritoneal circulation, vascular Endothelial Growth Factor (VEGF) and its receptors are key angiogenic factors involved in almost every step of peritoneal neovascularization. Angiogenesis is closely related to peritoneal fibrosis [49]. Damaged HPMC are the primary source of VEGF in the peritoneum [50]. Studies show that treatment with dapagliflozin can reduce VEGF levels in the peritoneal effluent [13], potentially leading to decreased neoangiogenesis. In the context of diabetic endothelial dysfunction, dapagliflozin may promote vascular repair by lowering the expression of vascular adhesion molecules and reducing inflammatory macrophage infiltration [51]. Collectively, these effects on the endothelium may contribute to the broader cardiovascular protection offered by SGLT-2 inhibitors [52].

### 3.4 Improving mitochondrial function and energy metabolism

Both locally in the peritoneum and systemically in the heart and kidneys, emerging evidence implicates mitochondrial damage in peritoneal injury during PD. At the peritoneal level, mitochondrial dysfunction can trigger inflammation in mesothelial cells, amplifying cytokine-induced responses through ROS generation and NF-κB activation [53]. SGLT-2 inhibitors can repair mitochondrial oxidative phosphorylation function and reduce ROS accumulation by activating the AMPK/PGC-1α pathway [54], [55]. SGLT-2 inhibitors can upregulate AMPK phosphorylation in peritoneal cells and restore the activity of mitochondrial complexes [56]. Specifically, empagliflozin has been found to increase functional mitochondrial mass, lower the Drp1/Mfn1 ratio (indicating reduced fission), decrease 8-OHdG levels (a marker of oxidative DNA damage), and reduce both mitochondrial and cytoplasmic ROS production [57].

Beyond the peritoneum, SGLT-2 inhibitors improve myocardial energy metabolism by promoting the heart's utilization of ketone bodies, thereby enhancing cardiac efficiency [58]. Additionally, SGLT-2 inhibitors can inhibit Na+/H+ exchange, increase ketone production, and lower intracellular myocardial Na+ and Ca2+ concentrations. The reduction in sarcoplasmic calcium leads to increased ventricular contractility, preventing harmful cardiac remodeling and ischemia-reperfusion injury [59], [60], [61].

### 3.5 Inhibiting inflammatory responses

Chronic inflammation during PD contributes significantly to peritoneal structural and functional damage [62]. Macrophages exist in two immunological types: M1 and M2. M1 promotes the secretion of IL-1β, IL-6, and TNF-α by macrophages, maintaining a chronic inflammatory state; conversely,M2 macrophages exhibit anti-inflammatory effects by secreting IL-1 receptor antagonist, IL-10, and collagen [63]. Recent studies highlight the anti-inflammatory properties of SGLT-2 inhibitors, demonstrating their capacity to strongly promote macrophage polarization toward the protective M2 phenotype [63], [64], and regulate M1/M2 infiltration under various pathological conditions [65]. Furthermore, experiments have shown that SGLT2 inhibition can prevent inflammation by suppressing macrophage accumulation and MCP-1 expression [66].

Notably, insulin can exert pro-inflammatory effects on peritoneal macrophages by increasing glucose uptake and fueling inflammatory pathways [67]. The experiment showed that feeding physiologically increased the number of peritoneal macrophages that secrete IL-1β in a glucose-dependent manner. Subsequently, IL-1β contributes to postprandial stimulation of insulin secretion. SGLT-2i, however, can prevent postprandial inflammation, thereby alleviating hyperglycemia and insulin release [68]. Empagliflozin, for instance, has been shown to lower levels of pro-inflammatory and pro-fibrotic markers like TGF-β, NF-κB, and caspase 3 [69].

Table 1 summarizes available clinical studies evaluating SGLT-2 inhibitors in patients undergoing peritoneal dialysis, highlighting the currently limited but emerging evidence base.

**Table 1:** Clinical studies of SGLT-2 inhibitors in patients undergoing peritoneal dialysis.

Study	Model	SGLT-2 inhibitors	Results	Visualized benefits
Lai JW et al., 2024 [70]	50 diabetic chronic PD patients	/	Ultrafiltration rate (1322 ± 200 ml/day vs. 985 ± 415 ml/day, p = 0.013)	Increase ultrafiltration volume
Schricker S et al., 2022 [19]	PD human peritoneal biopsies	/	The protein expression of SGLT-2 increases with the duration of PD and is significantly enhanced in patients with EPS.	Identifies SGLT-2 expression in the human peritoneum, elevated in EPS
Barreto J et al., 2023 [71]	7 patients with Kidney Failure and 7 DMP	dapagliflozin	Dapagliflozin C max was 117 and 97.6 ng/ml in the kidney failure and control groups, whereas the corresponding accumulation ratios were 26.7% and 9.5%.	Dapagliflozin has non-accumulating pharmacokinetic characteristics.
Hamdan Z et al., 2024 [13]	20 CAPD patient	dapagliflozin	Dapagliflozin was associated with a small increase in sodium dip, a decrease in peritoneal VEGF, and a decrease in systemic IL-6 levels	Dapagliflozin may reduce inflammation, inhibit angiogenesis, and protect the peritoneum.
Moral Berrio E et al., 2024 [72]	31 NDMP and DMP	empagliflozin and dapagliflozin	Both diabetic and non-diabetic patients maintained RKF and diuresis during treatment with SGLT-2 inhibitors. Albuminuria was significantly reduced in the diabetic group. SBP was also significantly reduced in the sample.	preserve RKF and diuresis, control blood pressure, and reduce proteinuria and weight.
Lai JW et al., 2023 [73]	DKD-5 stage (3 patients) and IgA nephritis (1 patient)	dapagliflozin	Case 1: Ultrafiltration volume increased from ±156 mL to 1260 mL. Case 2: Ultrafiltration volume increased from 740 ± 322 mL to 1140 ± 245 mL. Case 3: Ultrafiltration volume increased from ±220 mL to 1485 mL. Case 4: Ultrafiltration volume increased to 1267 ± 288 mL.	SGLT-2 inhibitors may increase ultrafiltration in newly initiated PD patients.

Abbreviations: PD, peritoneal dialysis; EPS, encapsulating peritoneal sclerosis; DMP, diabetic patients; NDMP, non-diabetic patients; CAPD, continuous ambulatory peritoneal dialysis; RKF, residual kidney function; SBP, systolic blood pressure; DKD, diabetic kidney disease; VEGF, vascular endothelial growth factor; IL-6, interleukin-6.

## 4 Potential benefits of SGLT-2 inhibitors in peritoneal dialysis

### 4.1 Local peritoneal benefits

#### 4.1.1 Improving peritoneal ultrafiltration

At the peritoneal membrane level, a novel hypothesis suggests that SGLT-2 inhibitors may limit glucose absorption via SGLT-2 transporters in the peritoneum itself, helping to maintain the osmotic gradient and preserve ultrafiltration capacity [20], [73]. The EMPOWERED trial, a randomized, double-blind, placebo-controlled crossover study initially designed based on preliminary data suggesting a potential 90 mL increase in ultrafiltration, ultimately found that empagliflozin did not significantly improve ultrafiltration volume in PD patients (−38 mL; 95% CI: −120 to 44; P = 0.36) [74]. Additionally, a case series of four incident PD patients demonstrated marked increases in ultrafiltration volume (ranging from 1140 to 1485 ml/day) following dapagliflozin initiation, compared with baseline values of 156–740 ml/day [73]. These findings suggest a potential benefit of SGLT-2 inhibitors on peritoneal fluid removal, although the current evidence is derived from observational data and case reports, and larger prospective studies are warranted to confirm these observations.

#### 4.1.2 Alleviating peritoneal fibrosis

Locally within the peritoneal cavity, SGLT-2 inhibitors have demonstrated anti-fibrotic effects through multiple pathways. By inhibiting TGF-β/Smad signaling, these agents suppress EMT in mesothelial cells [37]. Animal models have shown reduced dialysate-induced peritoneal thickening and collagen deposition following SGLT-2 inhibitor treatment [35], [75]. These effects may prolong technique survival by preserving peritoneal membrane integrity.

#### 4.1.3 Reducing peritoneal angiogenesis

Within the peritoneal microcirculation, damaged HPMC are the primary source of VEGF in the peritoneum [50]. Studies show that treatment with dapagliflozin can reduce VEGF levels in the peritoneal effluent, potentially leading to decreased neoangiogenesis [13], [76]. This effect may indirectly reduce peritoneal fibrosis, as angiogenesis is closely related to fibrotic changes [49].

## 5 Systemic cardio-renal and metabolic benefits

### 5.1 Improving metabolic parameters

Systemically, SGLT-2 inhibitors provide multiple metabolic benefits that are particularly relevant to PD patients. (1) Glycemic control: SGLT2 inhibition represents a novel insulin-independent pharmacological strategy for lowering blood glucose [77], [78]. **(2) Blood pressure reduction:** A meta-analysis showed that SGLT-2 inhibitors cause an average reduction of 3.62/1.70 mmHg in 24-h ambulatory systolic/diastolic blood pressure [79]. The initiation of dialysis leads to an early improvement in hypertension associated with end-stage renal disease. Subsequently, blood pressure control steadily deteriorates over time, which may be related to the late decline in peritoneal ultrafiltration and fluid retention [80]. **(3) Uric acid lowering:** Multiple clinical trials have demonstrated the serum uric acid-lowering effect of SGLT-2 inhibitors [81]. A retrospective analysis of data from 9405 peritoneal dialysis patients demonstrated that higher uric acid levels are an independent risk factor for all-cause mortality in peritoneal dialysis patients [82]. The mechanism is thought to involve SGLT-2 inhibitors inhibiting GLUT9 activity, thereby reducing uric acid reabsorption and increasing its urinary excretion [83], [84]. **(4) Weight management:** Meta-analyses demonstrate that SGLT-2 inhibitors can induce modest reductions in body weight and waist circumference [85]. This effect is attributed to calorie loss from glucosuria and may also involve reductions in ectopic fat deposition [86].

### 5.2 Preserving residual kidney function

By competitively inhibiting glucose and sodium reabsorption in the proximal tubule, SGLT-2 inhibitors increase distal sodium delivery to the macula densa. This triggers tubuloglomerular feedback (TGF), leading to afferent arteriolar constriction and reduced intraglomerular pressure, thereby alleviating glomerular hyperfiltration [24].

Residual kidney function (RKF) is a critical factor for both technique and patient survival [87]. Emerging real-world data suggest that SGLT-2 inhibitor use in PD patients is safe and effective for preserving RKF, likely by reducing glomerular hyperfiltration and proteinuria [72], [88]. These findings align with the landmark DAPA-CKD trial, which demonstrated significant renal protection with dapagliflozin in patients with CKD, including those with advanced disease [88].

### 5.3 Protecting cardiac function

At the systemic cardiovascular level, SGLT-2 inhibitors provide robust cardiac protection through multiple mechanisms. (1) **Antioxidant stress:** SGLT-2 inhibitors, particularly empagliflozin, have demonstrated antioxidant properties. Studies show that empagliflozin can reduce myocardial palmitic acid oxidation, thereby inhibiting oxidative stress in the heart [89], [90], [91]. (2) **Improving cardiac remodeling:** The PREDOMINACE trial, for instance, showed that dapagliflozin prevented early ventricular dysfunction and remodeling after myocardial infarction [92]. **(3) Reducing cardiovascular events:** A recent meta-analysis of 11 cardiovascular outcome trials involving 77,541 patients demonstrated reduced mortality and hospitalization rates, independent of T2D status [93]. SGLT-2 inhibitors may inhibit adverse cardiac remodeling and improve diastolic function by ameliorating endothelial dysfunction [94], thereby protecting the heart.

## 6 Potential adverse events of SGLT-2 inhibitors

While generally well-tolerated, the use of SGLT-2 inhibitors is associated with specific adverse events that warrant consideration, particularly in the vulnerable PD population.

### 6.1 Diabetic ketoacidosis (DKA)

An increased risk of euglycemic diabetic ketoacidosis (EDKA) has been recognized with SGLT-2 inhibitor use [95]. The mechanism involves drug-induced glucosuria and osmotic diuresis, leading to carbohydrate deficiency and an elevated glucagon/insulin ratio. This shift promotes lipolysis and free fatty acid oxidation, resulting in increased ketogenesis [96], [97]. Elevated levels of adrenaline and glucagon further enhance insulin resistance, ketogenesis, and lipolysis, leading to ketoacidosis [97], [98].

Due to the less severe insulin deficiency induced by SGLT2i, glucose excess and underutilization are not as prominent in quantity. This effect, coupled with the drug's inhibition of glucose reabsorption in the kidneys, may explain why plasma glucose in patients with SGLT2i-induced ketoacidosis is only slightly elevated [97]. While the absolute risk appears low (e.g., 0.2% in one hospitalized cohort), it is significantly higher compared to other drug classes like DPP-4 inhibitors [95], [99].

### 6.2 Genitourinary infections

Potential adverse events induced by SGLT-2 inhibitors commonly include urinary tract infections (UTIs) and genital mycotic infections (GMIs). The mechanism may be that SGLT-2 inhibitors reduce the reabsorption of glucose in the kidneys, thereby enhancing the excretion of glucose in the urine to lower blood sugar, thereby creating a significantly favorable environment for bacterial growth [100]. Recently, many studies have proven the association between SGLT-2 inhibitors and UTIs. An analysis based on the FAERS database revealed a strong correlation between SGLT-2 inhibitors and UTIs and GMI [101]. Factors such as a history of genital mycotic infections and/or UTIs may limit their applicability in certain patients.

#### 6.2.1 Urinary tract infections

There is much debate surrounding UTIs associated with SGLT-2 inhibitors. Pooled data from 12 double-blind controlled clinical trials of dapagliflozin evaluated the effect of pharmacologically induced glycosuria on the incidence of UTIs. Compared to placebo, the incidence of clinically diagnosed UTIs was slightly higher in female patients treated with dapagliflozin [102]. Another retrospective analysis proved that clinical symptoms and laboratory signs of UTIs were more common in patients receiving SGLT-2i treatment. In patients receiving SGLT-2i treatment, more than half of the urine cultures were positive [103]. Although we still cannot fully understand this mechanism, we should also be more cautious when using SGLT-2 inhibitors.

#### 6.2.2 Genital mycotic infections

GMIs are more consistently reported, with incidence rates in clinical trials ranging from 2.3% to 13.4% [104]. Case reports, such as *Candida glabrata* urinary tract infections, have been documented, with resolution upon drug discontinuation [105]. A history of such infections may limit the use of SGLT-2 inhibitors in some patients.

### 6.3 Peritonitis and volume status in PD patients

In addition to the adverse events observed in the general population, PD patients face unique safety considerations. Peritonitis remains a major complication in PD, and although no direct evidence links SGLT-2 inhibitors to an increased risk of peritonitis, careful monitoring is warranted given the immunosuppressed state and increased infection susceptibility in this population. Encouragingly, a large real-world study of 29,529 PD patients with type 2 diabetes found that SGLT-2i use was associated with a significantly lower risk of PD-associated peritonitis (aHR 0.340), without increasing the risk of volume depletion or genital infections [106]. Furthermore, the osmotic diuretic effect of SGLT-2 inhibitors may affect volume status and ultrafiltration in PD patients. However, available data from PD populations have not demonstrated an increased risk of hypotension or volume depletion, and a case series of incident PD patients observed no hypotension episodes after dapagliflozin initiation [73]. Close monitoring of fluid balance, blood pressure, and ultrafiltration volumes is nonetheless recommended, particularly during treatment initiation and dose titration.

### 6.4 Fractures and amputations

Currently, data on fractures and amputations caused by SGLT-2 inhibitors are limited, but recently a meta-analysis of 40 cohort studies, including 9,911,454 patients, proved that SGLT-2i was similarly associated with the incidence of lower limb amputations (LLA) and fractures compared to other hypoglycemic agents. Compared with other hypoglycemic agents, canagliflozin specifically increased the risk of LLA [107]. However, there is also contrary evidence that SGLT-2 inhibitors are not significantly associated with fractures and amputations, and even a small study has proven that the SGLT-2 inhibitor empagliflozin can significantly improve glucose metabolism in patients with diabetic osteoporosis, increase blood calcium and phosphorus concentrations, help reduce the incidence of fractures and fracture risk, and improve quality of life [108]. Given the uncertainty, caution is advisable, particularly in patients with pre-existing osteoporosis or high fracture risk.

### 6.5 Considerations for drug interactions

PD patients often require polypharmacy, increasing the potential for drug interactions. SGLT-2 inhibitors may interact with other antidiabetic agents, diuretics, and antihypertensive drugs, potentially affecting efficacy and safety. For instance, concomitant use with diuretics may potentiate volume depletion, while combination with insulin or sulfonylureas increases hypoglycemia risk. Careful medication review, patient education, and regular monitoring are essential to mitigate these risks and optimize treatment outcomes.

## 7 Conclusion

Long-term PD, while life-sustaining for kidney failure patients, can lead to peritoneal damage and ultimately dialysis failure, primarily driven by fibrosis. The advent of SGLT-2 inhibitors offers a promising new therapeutic avenue for this population. Locally, these agents may preserve peritoneal membrane integrity by reducing fibrosis, EMT, and neoangiogenesis, while improving ultrafiltration capacity. Systemically, they provide robust cardio-renal protection through metabolic improvement, RKF preservation, and cardiovascular risk reduction. Their documented anti-fibrotic, anti-inflammatory, and antioxidant effects, coupled with their ability to improve mitochondrial function, protect the vascular endothelium, and enhance cardio-renal outcomes, position them as agents with significant potential to prolong technique survival and reduce adverse events in PD patients. However, their use is not without potential risks, including DKA and infections. A judicious approach, weighing individual patient characteristics against indications and contraindications, is crucial to maximize their efficacy and safety in clinical practice.

## 8 Future prospects

Future research must prioritize dedicated, randomized controlled trials in PD patients to evaluate the long-term efficacy and safety of SGLT-2 inhibitors. Key endpoints should include their impact on RKF preservation, peritoneal membrane function, cardiovascular event rates, peritonitis risk, volume status, and overall survival. In addition, further research is needed to elucidate the mechanisms and protective effects of SGLT-2 inhibitors from multiple perspectives. With advancements in science and technology, there is great potential for a better understanding of the underlying mechanisms of peritoneal fibrosis and the identification of new biomarkers and therapeutic targets. Research also needs to consider individual patient differences, such as underlying conditions, comorbidities, and drug interactions, to determine the optimal use of SGLT-2 inhibitors. The development of clinical practice guidelines specifically addressing SGLT-2 inhibitor use in PD patients would be a significant step forward, providing clinicians with evidence-based recommendations for this growing patient population.

In conclusion, the intersection of SGLT-2 inhibitor therapy and peritoneal dialysis represents a dynamic and promising field of investigation with the potential to significantly improve outcomes for a growing number of patients with kidney disease.

## CRediT authorship contribution statement

**Yanling Song:** Writing – original draft, Visualization, Software, Resources. **Xiaofei Huang:** Software, Resources, Investigation, Formal analysis. **Weijie Yuan:** Writing – review & editing, Supervision, Software. **Nan Zhu:** Writing – review & editing, Project administration, Methodology, Funding acquisition, Conceptualization.

## Ethics approval and consent to participate: Not Applicable

Consent for publication: Not Applicable.

## Funding

This project was supported by the Clinical Research Innovation Plan of Shanghai General Hospital.

## Declaration of competing interest

The authors declare the following financial interests/personal relationships which may be considered as potential competing interests: Zhu nan reports financial support and administrative support were provided by Shanghai Jiao Tong University School of Medicine Affiliated First People's Hospital. ZHUNAN reports a relationship with Shanghai Jiao Tong University School of Medicine Affiliated First People's Hospital that includes:. Given his/her/their role as, had no involvement in the peer review of this article and had no access to information regarding its peer review. Full responsibility for the editorial process for this article was delegated to another journal editor. If there are other authors, they declare that they have no known competing financial interests or personal relationships that could have appeared to influence the work reported in this paper.

## Data Availability

No data was used for the research described in the article.
